# Real Life Population Pharmacokinetics Modelling of Eight Factors VIII in Patients with Severe Haemophilia A: Is It Always Relevant to Switch to an Extended Half-Life?

**DOI:** 10.3390/pharmaceutics12040380

**Published:** 2020-04-21

**Authors:** Quentin Allard, Zoubir Djerada, Claire Pouplard, Yohann Repessé, Dominique Desprez, Hubert Galinat, Birgit Frotscher, Claire Berger, Annie Harroche, Anne Ryman, Claire Flaujac, Pierre Chamouni, Benoît Guillet, Fabienne Volot, Jean Szymezak, Philippe Nguyen, Yoann Cazaubon

**Affiliations:** 1 Department of Medical Pharmacology, University Hospital of Reims, EA3801, SFR Cap-Santé, University of Reims, 51100 Reims, France; quentin.allard@bioardaisne.fr (Q.A.); zoubir.djerada@univ-reims.fr (Z.D.); 2 Department of Haemostasis, University Hospital of Tours, 37000 Tours, France; claire.pouplard@univ-tours.fr; 3 Department of Haemostasis, University Hospital of Caen, 14000 Caen, France; repesse-y@chu-caen.fr; 4 Department of Haemostasis, University Hospital of Strasbourg, 67000 Strasbourg, France; dominique.desprez@chru-strasbourg.fr; 5 Department of Haemostasis, University Hospital of Brest, 29200 Brest, France; hubert.galinat@chu-brest.fr; 6 Department of Haemostasis, University Hospital of Nancy, 54000 Nancy, France; b.frotscher@chru-nancy.fr; 7 Department of Haemostasis, University Hospital of Saint-Etienne, 42000 Saint-Etienne, France; claire.berger@chu-st-etienne.fr; 8 Department of Haemostasis, Hospital Necker, Assistance Publique-Hôpitaux de Paris, 75015 Paris, France; annie.harroche@aphp.fr; 9 Department of Haemostasis, University Hospital of Bordeaux, 33000 Bordeaux, France; anne.ryman@chu-bordeaux.fr; 10 Department of Haemostasis, Hospital of Versailles, 78000 Versailles, France; cflaujac@ch-versailles.fr; 11 Department of Haemostasis, University Hospital of Rouen, 76000 Rouen, France; Pierre.Chamouni@chu-rouen.fr; 12 Department of Haemostasis, University Hospital of Rennes, 35000 Rennes, France; Benoit.GUILLET@chu-rennes.fr; 13 Department of Haemostasis, University Hospital of Dijon, 21000 Dijon, France; fabienne.volot@chu-dijon.fr; 14 Department of Haemostasis, University Hospital of Reims, EA3801, SFR Cap-Santé, University of Reims, 51100 Reims, France; jszymezak@chu-reims.fr (J.S.); pnguyen@chu-reims.fr (P.N.)

**Keywords:** factor VIII, severe haemophilia A, pharmacokinetics, switch, modelling, dose tailoring

## Abstract

We retrospectively analysed the data files of 171 adults and 87 children/adolescents with severe haemophilia, except for 14 patients (moderate; minor) (1), to develop a global population pharmacokinetic (PK) model for eight factors VIII (FVIII) that could estimate individual PK parameters for targeting the desired level of FVIII activity (FVIII:C); and (2) to compare half-life (HL) in patients switching from a standard half-life (SHL) to an extended half-life (EHL) and evaluate the relevance of the switch. One-stage clotting assay for the measurement of FVIII activity (FVIII:C, IU/mL) was used for population PK modelling. The software, Monolix version 2019R1, was used for non-linear mixed-effects modelling. A linear two-compartment model best described FVIII:C. The estimated PK parameters (between-subject variability) were: 2640 mL (23.2%) for volume of central compartment (V1), 339 mL (46.8%) for volume of peripheral compartment (V2), 135 mL/h for Q (fixed random effect), and 204 mL/h (34.9%) for clearance (Cl). Weight, age, and categorical covariate EHL were found to influence Cl and only weight for V1. This model can be used for all of the FVIII cited in the study. Moreover, we demonstrated, in accordance with previous studies, that Elocta had longer half-life (EHL) than SHL (mean ratio: 1.48) as compared to Advate, Factane, Kogenate, Novoeight, and Refacto.

## 1. Introduction

Haemophilia A is a genetic coagulation pathology. It is manifested by bleeding, secondary to the deficit of one of the factors of coagulation: factor VIII (FVIII). The treatment of haemophilia A is based on supplementation, prophylactic, or on-demand, in FVIII. Since the development of these treatments in the 1950s, the quality of life and life expectancy of haemophilia A patients improved and they are tending, in developed countries, to approach those of the non-affected population [1,2]. Currently, there is no optimal prophylactic regimen for all patients. Despite an adaptation of dose according to weight, the high interindividual variability (IIV) of FVIII pharmacokinetics (PK) makes it difficult to retain the coagulating activity of FVIII (FVIII: C) above a targeted level [3]. It is essential to improve patient safety while optimising the use of FVIII [4]. An under-dose of FVIII exposes the patient to a risk of bleeding, whereas overdose leads to the excessive use of resources without therapeutic benefit [5,6]. Ideally, the patient’s PK profile is taken into account and is scheduled in advance to develop an optimised dosing regimen [7]. The main obstacles to this approach in current clinical practice are the complex calculations that are involved and a large number of samples required during the first 72 h [8,9]. This last problem can be overcome by using the pharmacokinetic of population (PKPOP) approach, reducing the number of samples required for a precise estimation of the PK parameters [3]. PKPOP approach allows for quantifying the interindividual variability of the estimated PK parameters. Knowing the particularities linked to FVIII makes it possible to explain part of the variability by testing the covariates having a biological meaning (e.g., age, weight, von Willebrand Factor, ABO blood group). Once qualified, the model becomes able to determine the PK parameters of the individual and, thus, personalise the dose administered according to the frequency of administration, individual covariates, and desired target reassessed based on patient’s life context. Thus, the optimised prophylactic regimen for each patient that allows for maintaining FVIII:C above its targeted threshold can be more effectively determined [10].

The objectives of this study were to develop a global population PK model in patients with severe haemophilia A and compare half-life (HL) between patients switching from standard half-life (SHL) to extended half-life (EHL).

## 2. Materials and Methods

### 2.1. Study Design, Subjects, Sample Collection and Assay Method

This retrospective multicentre (*n* = 13) study was a characterisation of interindividual PK variability of data collected between 2012 and 2019 from patients treated with FVIII concentrates for mainly severe haemophilia A. The data were taken from current practice. An individual information note was distributed to patients to ensure that they were not opposed to participating in this study. Thus, the database is in compliance with the reference methodology MR004 of the “Commission Nationale de l’Informatique et des Libertés”. All of the patients had severe, moderate, or minor haemophilia A and did not present inhibitors during PK analysis.

FVIII:C was measured using the one-stage clotting assay principle. For FVIII:C measurement, the couple, reagent kit-coagulation analyser, varied according to the centres: STA R-STA-CK Prest (*n* = 8), STA R-Pathromtin (*n* = 1), and ACL TOP-SynthAsil (*n* = 4). The blood samples were collected at different times, before infusion (predose) and between 15 min. and 96 h after infusion. The number of instances of blood collection varied between one and 11 samples per patient (median: 4). The lower limit of quantification (LLOQ) was different among centres: 0.004 and 0.01 IU.mL^−1^ (median: 0.01 IU.mL^−1^). The percentage of FVIII:C below LLOQ (BLQ) of the dataset was 10.6%. Table 1 shows the details of the modelling dataset.

### 2.2. PK Analysis

Population PK analysis of FVIII:C was conducted while using the nonlinear mixed-effects modelling approach that was implemented in Monolix software (version 2019R1, Lixoft, Antony, France, http://lixoft.com/) using the Stochastic Approximation Expectation Maximisation (SAEM) algorithm [11,12]. All of the individual PK parameters were assumed to be log-normally distributed. Exponential random effects were used to describe between-subject variability (BSV). Data that were below LLOQ (BLQ) were handled in a right-truncated Gaussian distribution while using the SAEM algorithm [13]. To take endogenous and predose FVIII:C (corresponding to the residual before the PK data dose) into account, PK modelling was done at a steady-state. Because we know the time interval between the dose corresponding to predose and the PK data dose, we virtually added four doses before these two last doses (respecting this interval administration) to ensure steady-state for all individuals. When predose was not known, BLQ (<0.01 IU/mL) data were assumed for PK modelling (uniquely for severe haemophilia A patients. For moderate and minor haemophilia A patients, the predose was known). Moreover, we tested another approach to estimate endogenous FVIII production with an endogenous production rate included in the structural PK model [14].

#### 2.2.1. First Step—Basic Model Building

One-, two-, and three-compartment models with first-order elimination were initially compared. Several error models (constant, proportional or combined error model) were assessed for describing the residual variability (ε). We split analysis depending on the reagent used to select the best error model because the reagents and coagulation analyser differed between the centres for the measurement of FVIII:C.

#### 2.2.2. Second Step—Covariate Analysis

From the basic model (without covariate), the effects of five covariates on FVIII:C PK parameters were evaluated: age, total body weight (TBW), brand, structure of FVIII (categories: fc Fusion, full-length recombinant, B-domain deleted and plasma-derived), and EHL (elocta:1 versus others:0). We also tested the von Willebrand Factor (vWF), ABO blood group and fat-free mass (FFM) [15] to explore the impact of these covariates, but we decided not to include them in the final model because of missing covariates for few individuals, as follows: 130 for vWF, 51 for ABO group and 61 for FFM.

For continuous covariates, the parameter-covariate relationships were modelled, as follows:

(1) $${CL}_{i}={CL}_{pop}\times {\left(\frac{{COV}_{i}}{{COV}_{median}}\right)}^{\beta }\times e^{\eta_{CL,i}},$$

where β is the regression coefficient to be estimated, *COV_i_* is the covariate value for subject *i*, and *COV_median_* is the median value of the covariate in the study population.

For categorical covariates, the general equation was:

(2) $${CL}_{i}={CL}_{pop}\times e^{\beta .COVi}\times e^{\eta_{CL,i}},$$

where *COV_i_* is 0 or 1 (0: others; 1: Elocta).

The covariate model was built while using a stepwise procedure with forwarding inclusion and backward deletion. Preliminary to the covariate selection process, a graphical inspection was done to assess the correlation between individual parameters and random effects versus covariates. A covariate was kept in the model if it improved the fit, reduced interpatient variability, and decreased the objective function (corresponding to -2log likelihood) and Bayesian information criterion (BIC). The statistical significance of covariate was evaluated while using the likelihood ratio test (LRT). The covariate was retained in the model if the LRT was reduced by at least 3.84 (χ2 *P* < 0.05 for one degree of freedom). Next, during the stepwise deletion from the full model, the covariate was statistically significant, with an increased LRT of 10.83 (χ2 *P* < 0.001 for one degree of freedom). Finally, the Wald test (stochastic approximation) regarding individual parameters versus covariates had to be less than *P* < 0.05 to keep the covariate in the final model.

### 2.3. Internal Evaluation of the Model

The evaluation of the model was based on goodness-of-fit plots, that is, observations versus individual and populations predictions (OBS-PRED), individual weighted residuals versus individual predictions and time (IWRES), plots of normalised prediction distribution error (NPDE) versus population predictions and time [16], and prediction-corrected visual predictive checks (pcVPC) which were performed with 1000 simulated data sets [17]. pcVPC showed the time course of the 10th, 50th, and 90th percentiles with a 90% level of confidence around the simulated profiles and compared with observed data.

### 2.4. Model Qualification Process

The most appropriate pharmacostatistical model was selected and evaluated based on the following criteria: decrease of the objective function (calculated by importance sampling) and BIC; inspection of the usual diagnostic plots (OBS-PRED, IWRES, NPDE, pcVPC); a decrease of BSV and a low relative standard error (RSE < 30%) of PK parameter estimates.

In the final model, the 95% confidence interval of each parameter was determined from 1000 nonparametric bootstraps based on resampling [18,19] while using R package Rsmlx [20].

All of the runs were performed five times using the convergence assessment tool of Monolix, which consisted of different randomly generated initial values of fixed effects as well as different seeds to assess the robustness of the convergence.

### 2.5. External Comparison of Half-Life Estimates Between Our Model and Mc Eneny-King SHL Model [21]

Mc Eneny-King et al. [21] developed a generic population PK model for SHL FVIII. They pooled in their model seven FVIII: Advate, Kovaltry, Kogenate, Novoeight, Refacto AF, Emoclot, and Octonate. Each FVIII was represented by at least 30 PK, except for Emoclot (*n* = 14). We implemented their model in Monolix software in accordance with the structure of the model by fixing all population parameters, omegas, and error model values. The dataset without missing covariates contained 197 patients because one of the covariates of their model was FFM. Subsequently, the individual PK parameters were estimated and extracted (mode of empirical bayes estimates, EBEs) to calculate HL of each individual and compared to our model.

### 2.6. HL Comparison after Switch

The dataset contained switched PK SHL to EHL of 44 patients (a total of 88 patients considering two different patients for each pair). We compared the elimination half-lives of patients who switched from Advate, Factane, Kogenate, Novoeight, or Refacto to Elocta. To do this, we extracted from our qualified final model the mode of the EBEs, which are the most probable values of the individual parameter estimates.

The association between different predictors as switching from SHL to Elocta, age and weight, and half-life were assessed while using multivariate analysis using multiple linear regression analysis. The stepping method, with *p* < 0.05 for entry and *p* < 0.01 for removal, was used for predictor selection. Different assumptions were checked on the residues to test the validity of the linear regression model: (1) the minimum and maximum values of the standardised residual are within [−3, +3] values; (2) the data points are independent and tested by the Durbin–Watson test; and, (3) the distribution of the standardised residuals should be normal, with mean = 0 and a constant variance not different from 1, and graphically by means of a histogram, scatterplot, and Q–Q plot. Multicollinearity was checked while using collinearity statistics (variance inflation factor of approximately 1). Final multiple linear model was externally validated, using the cross-validation technique [19]. Multivariate analysis was performed with R 3.1.4 (The R Foundation for Statistical Computing, http://www.r-project.org).

## 3. Results

### 3.1. Subject Characteristics

A total of 258 patients (87 children and 171 adults), corresponding to 935 plasma concentrations, were included for model development on the condition that the date of the therapeutic initiation would be known with at least one sample, excluding residual activity. Among the 258 patients used for model development, 44 patients switched from SHL to EHL (a total of 88 patients considering two different patients for each pair). Table 1 provides the demographic characteristics of the study population.

### 3.2. Basic Model Building

A two-compartment model with linear elimination was identified as the best model for describing the PK of FVIII:C of eight brands. Despite variability for activated partial thromboplastin time (APTT) reagent and the coagulation analyser (STA R-STA-CK Prest, STA R-Pathromtin, ACL TOP-SynthAsil), the more appropriate error model was the combined error model (see Figure S1), because its BIC was the smallest (data not shown) as compared to that for the additive or proportional error model. The combined error model is represented by the following formula:

(3) y=Cc+(a+b×Cc)×e

where *y* corresponds to the observations from the data set, *Cc* to the predicted observations (output of the structural model), *a* and *b* to the parameters of the residual error model, and *e* to the variable randomly normal distributed generating the residual error. Estimation of endogenous FVIII production by including an endogenous production rate in the structure did not improve the capability of model prediction (ΔBIC > 0 compared to final model). By applying parsimonious principle, we decided not to keep this kind of structure for the final model.

### 3.3. Covariate Analysis

Continuous covariates were centered on the median of the population dataset. Significant EHL (others: 0, Elocta: 1), age, and weight effects were observed on the Clearance (Cl), and weight effects were observed on the central volume of distribution (V1). These covariates were included in the final model and were associated with a reduction in the unexplained IIV of Cl from 45.3% to 34.9% and of V1 from 45.6% to 23.2%. βweight on Cl corresponds to the regression coefficient. The weight regression coefficient was fixed at 0.75 for Cl, because it was in the confidence interval of the estimated one (median value: 0.779, RSE: 9%) [22]. We decided not to include the covariate Brand because of insufficient data for some brands (Factane, Kovaltry, Afstyla, Novoeight, Refacto) were causing overparametrisation and overfitting observed with high RSE of these brands (see Table S1.). For the covariate Structure, there was no significant difference among BIC, RSE, and decrease in *ω Cl*, so we applied the parsimonious principle. Moreover, concerning the covariate Structure, we calculated the HL, including either EHL covariate or FVIII Structure covariate on Cl. The difference was not statistically significant (see Figure S2). Table 2 summarises the PK parameters of the final model. All were reliably estimated, as reflected by the small RSEs from the observed Fisher information matrix. The results of bootstrap medians and 95% confidence intervals were consistent. The bootstrap analysis confirmed the reliability and robustness of the parameter and random effect estimates. Thus, the final model with covariates was representative.

Even though they are not part of our final qualified model, Table S2 presents the impacts of ABO, vWF, and FFM covariates on Cl and/or V1.

### 3.4. Internal Evaluation of the Final Model

**Figure 1:**
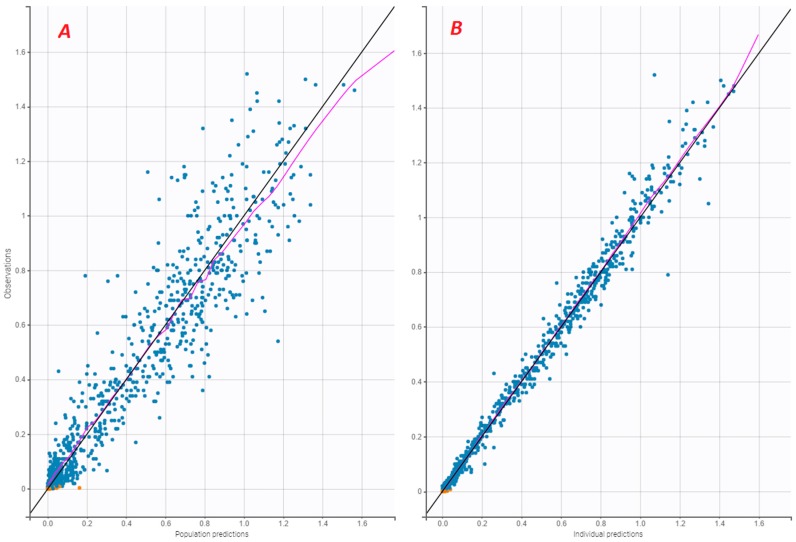
(**A**) Observed FVIII:C (IU/mL) versus population predictions (IU/mL); (**B**) Observed FVIII:C (IU/mL) versus individual predictions (IU/mL). Blue dots are observed FVIII:C; orange dots are observed FVIII:C BLQ; the purple solid line is the spline.

**Figure 2:**
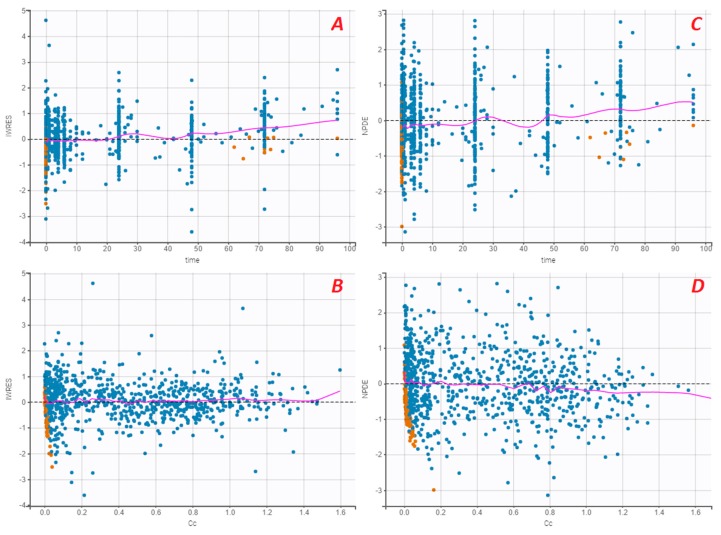
Individual weighted residual (IWRES) versus time (**A**) and individual predictions after dose (**B**). Normalised prediction errors (NPDE) versus time (**C**) and population predictions after dose (**D**). Abbreviations: Cc, FVIII:C (IU/mL); time in hour. Blue dots are observed FVIII:C; orange dots are observed FVIII:C BLQ; the purple solid line is the spline.

**Figure 3:**
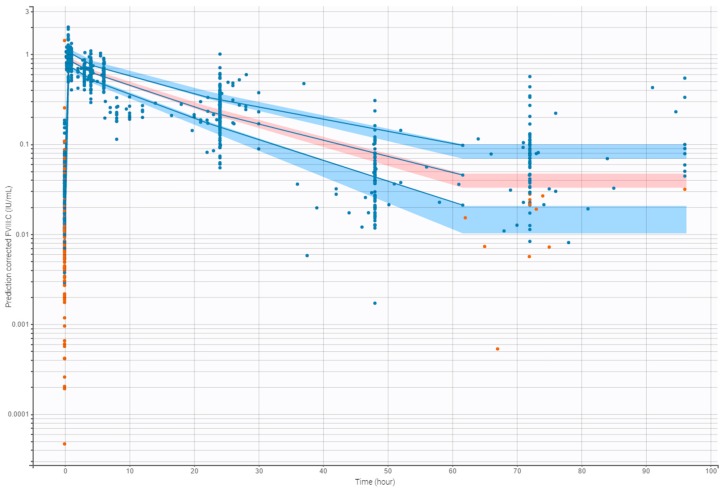
Prediction-corrected visual predictive check (pcVPC) for FVIII:C (IU/mL). Blue dots are observed FVIII:C (IU/mL); orange dots are observed FVIII:C BLQ; blue solid lines represent the median, 10th and 90th percentile of the observed values and shaded areas represent the spread of 90% prediction intervals calculated from simulations.

The standard error of the PK parameter estimates was less than 30% for the structural parameters, random effects, residual error, and correlation (V1, Cl). Figure 1, Figure 2 and Figure 3 present the goodness-of-fit plots of the final model. The observed and predicted concentrations of FVIII:C matched well by visualising individual weighted residual (IWRES) versus time after a dose and individual predictions (Figure 2). No major systematic bias was observed for NPDE (Figure 2). The pcVPC plot presented in Figure 3 indicated a good predictive performance of the model. Additionally, the model appeared to satisfactorily predict the median tendency and dispersion of the observations. Overall, the 10th, 50th, and 90th percentiles of the observed concentrations were within the predicted 90% confidence interval of these percentiles.

### 3.5. HL Comparison between Our Global Model and SHL Generic Model of McEneny-King et al. [21]

**Figure 4:**
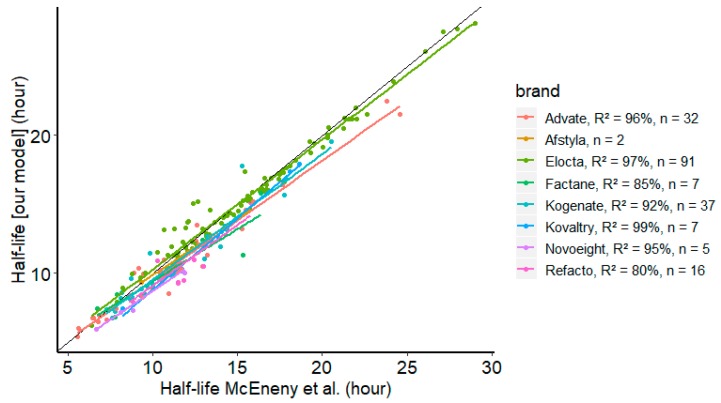
Comparison between estimated half-life from our model and McEneny-King’s et al.’s generic standard half-life (SHL) model [21].

**Figure 5:**
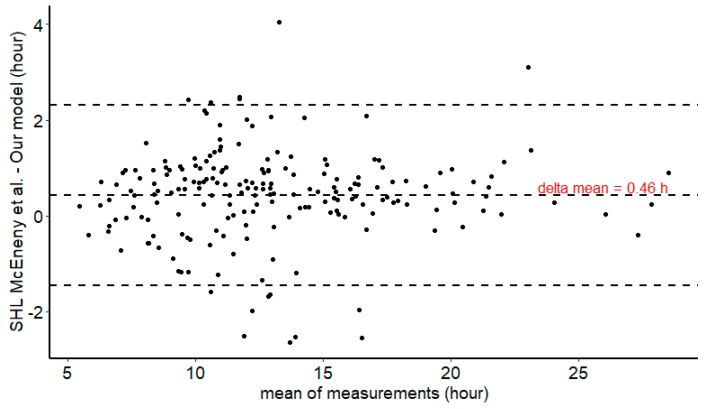
Bland Altman plot of half-life empirical bayes estimates (EBEs) of our model and McEneny-King’s et al.’s generic SHL model [21].

We compared the individual prediction capability of McEneny-King et al.’s model with our dataset to the individual prediction capability of our model. For most of FVIII, the half-life estimates were equivalent—the Refacto (Figure 4) was the exception—and showed a high correlation for the whole set of brands: *R*^2^ = 95%. When these two models are compared, Figure 5 allows for one to appreciate the delta (difference) between the estimates of HL.

### 3.6. HL Comparison after Switch

**Figure 6:**
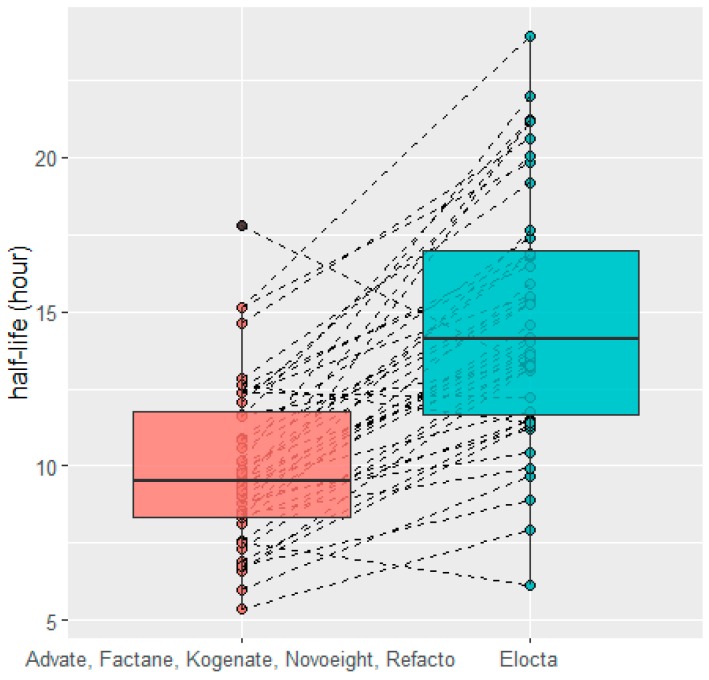
Half-life comparison before and after switching from Advate, Factane, Kogenate, Novoeight, or Refacto to Elocta.

Figure 6 shows the HL of FVIII:C when the patients switched from SHL to EHL. The HL was increased, on average, by a factor of 1.48. Of the 44 patients, four (9%) had a decrease in half-life at the time of the switch: −0.16, −1.21, −1.34, and −4.57 h. Using multivariate analysis, HL was associated, with the switching, by increasing the half-life by 4.8 ± 0.73 h (*p*-value < 0.05). Additionally, age increased HL by 0.13 ± 0.03 h·year^−1^ (*p*-value < 0.05) and weight decreased HL by 0.055 ± 0.024 h·kg^−1^ (*p*-value < 0.05). Table S3 presents the characteristics of patients and the ratio of HL, EHL/SHL.

## 4. Discussion

We developed and qualified a population PK model of FVIII:C from eight different forms of FVIII while using data provided from clinical practice. This work was original because it used real-life routine patient data while implementing the covariate EHL (0:SHL, 1:Elocta) and merging eight FVIII in the modelling process.

The population parameters were comparable to those that were estimated in the published generic SHL FVIII model [21]. Our model had a correlation between V1 and Cl and, despite the heterogeneity of ‘coagulation analyser-APTT reagent’, it presented a combined error model. This is in agreement with a few models found in the literature [3,23].

TBW explained a significant part of IIV on V1 and Cl. The covariate FFM was secondary tested on 197 patients among 258 (height not available for all). When comparing BSV and BIC, FFM is mathematically and physiologically better. It seems to be a good TBW substitute. BSV of V1 or Cl, when either TBW or FFM is included, seem to be quite similar (See Table S2). TBW is a good compromise because it is simple to obtain. Age was a significant covariate on Cl, as mentioned in the literature [3]. ABO blood group and vWF were also secondary tested to evaluate the potential of these covariates as plausible biomarkers in terms of FVIII physiology (not available for all patients included: 128 and 207 patients were measured for vWF and ABO blood group, respectively). We observed a positive correlation between age and vWF (*R*^2^ = 32%). The addition of the covariate ABO or vWF on Cl at the end of the modelling process showed a 3% or 6.1% decrease of BSV Cl when compared to the final model for each modelling process, respectively. Additionally, the covariate age became non-significant when these two covariates were added on Cl. Moreover, PKpop models included ABO or vWF as a significant covariate on Cl, confirming the relevance of their use [23,24,25,26,27]. Even if the vWF is subject to inter-occasion variability (i.e., surgery, inflammation), this covariate seems to be necessary for the characterisation of BSV Cl, and it reflects its protector role for FVIII. Therefore, it would be interesting to develop a global model with vWF while using data from patients developing inflammation or undergoing surgery.

The BSV for one FVIII seems to be equivalent to the BSV generated with many brands of FVIII, as mentioned by McEneny-King et al. [21]. This is confirmed by the fact that the inclusion of a covariate brand or structure did not significantly reduce the BSV. Moreover, our BSV Cl and V1 estimates were slightly weaker than those of their model, whereas our modelling dataset contained EHL. The difference between our population characteristics (age, TBW, number of patients) may explain this, given that age and TBW are significant covariates.

We externally compared the individual parameters predictions of our model while using those that were estimated with the generic SHL model of McEneny-King et al. [21]. The results confirmed that their model is capable of predicting individual brands outside the original database, EHL. This shows that the BSV of one factor VIII is equivalent to the BSV of different FVIII.

We then compared the data from patients undergoing a switch from SHL to Elocta, the ratio from the A-LONG study [28] HL Elocta/SHL was 1.53 and comparable with ours of 1.48. However, that study’s median HL for Elocta was greater than ours: 19 h versus 14.8 h. This is explained by our population, which had more children and especially children under 12 years old.

Bayesian tools allow for prediction with the precaution that a PK comparison should be carried out before switching to another FVIII to help clinicians/patients make an optimal FVIII choice. Indeed, it is not always justified to switch to an EHL. In our case, the probability of obtaining a decreased HL by switching from SHL to EHL was approximately 9%. However, for the remaining 91%, the benefit was significant in terms of improving patient comfort regarding the frequency of administration or elevation of FVIII:C (to be determined according to the patient’s lifestyle). Accordingly, it is necessary to evaluate the modification of the HL with a comparison of individual SHL versus EHL.

Our study has several limitations. First, 25% of patients had sparse PK data available. The brands Afstyla, Factane, Kovaltry, and Novoeight are underrepresented (for each cited brand, n_patient PK_ ≤ 10) to assert that this global model would do as well as a specific brand model, even if the criteria for evaluations agreed with its use. However, our model is in concordance with the McEneny-King model with a mean decreased half-life of 0.46 h for our model. This is reassuring in its use as global FVIII PK-tailoring with a downside on the Refacto, which seems to be described by a model structure with one-compartment [29]. We did not estimate endogenous FVIII production but we took into account predose (including endogenous part) by considering patients at steady-state. Moreover, our additive error (0.008 IU/mL) was closed to BLQ. By estimating endogenous FVIII production, it seemed to compromise the accuracy of error model estimate.

Population PK should be systematically integrated to improve medical decision making. In the context of high IIV, we can use this model to estimate the individual parameters. The rational manner to maintain FVIII:C above desired target and to improve visibility of physicians is to combine the samples at optimal times, as preconised by literature [3], and use this model for the optimisation of prophylactic dosing regimens in severe haemophilia A.

The immunogenicity of EHL is often under-evaluated post-marketing. Safety considerations when switching is mandatory, even if the probability of developing neutralising antibodies is very low in previously treated patients. A meticulous assessment of patients’ needs, an individual PK comparison, and cost/effectiveness may be required to inform a brand switch.

## 5. Clinical Application (Example)

**Figure 7:**
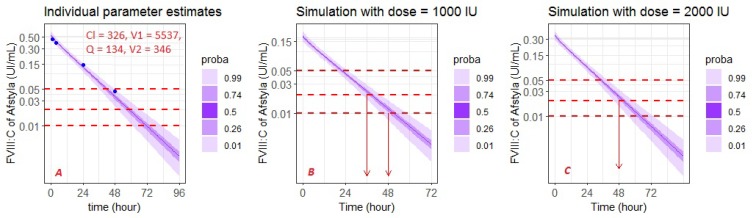
Estimated individual parameters (**A**) and rational adaptation dosing regimen of one individual (**B**,**C**). Blue dots are observed data.

Currently, tools for calculating individual patient parameters improve prediction control. In prophylaxis for haemophilia A, accurate estimation of HL and time-to-trough are crucial in reducing the risk of spontaneous bleeding. Sampling at optimal times using a population model is necessary to correctly estimate individual parameters [3] and minimise the risk of FVIII:C falling below the threshold. We selected a patient who was not included in the modelling dataset to show the process of individual parameter estimations (Figure 7A), and then used it to simulate adequate dosage using simulX (mlxR package) (Figure 7B,C). In our case, the patient (a 21-year-old man, 105 kg) took 3000 IU of Afstyla. He came for a consultation and wished to administer an injection every 48 h while being above the 0.01 IU/mL target. With 1000 IU, the patient had a risk of 1% of being under 0.01 IU/mL when FVIII was administered each 48 h. If a clinician estimates that the patient must have a time-to-trough threshold of 0.02 IU/mL because of his lifestyle, he will be covered for 36 h with 1000 IU and 48 h with 2000 IU.

The rational adaptation of this type of method is useful for calculating the optimal dose, allowing for an estimation of time above the desired target. Bayesian tools must be qualified to democratise this rational approach. By integrating these tools in a mobile app, the patient could also become actors in their own health by visualising their FVIII:C at time ‘t’ to increase their vigilance and readjust their behaviour according to their lifestyles.

## Figures and Tables

**Table 1:** Demographic characteristics and sampling information of the 258 patients included in the pharmacokinetic (PK) analysis.

Patients Characteristics	Median (Min-Max)
Age (year)	30 (3–77)
Weight (kg)	64 (15.1–130)
Haemophilia status	Severe, *n* = 244; moderate, *n* = 11; minor, *n* = 3
Treatment	Factane, *n* = 8; Advate, *n* = 44; Kogenate, *n* = 34; Kovaltry, *n* = 7; Afstyla, *n* = 5; Refacto, *n* = 18; Novoeight, *n* = 6; Elocta, *n* = 136
**Sampling Information**	
Number of BLQ samples (%)	99 (10.6%)
Number of samples per patient	4 (1–11)
Number of patients with one sample	63, no residual and FVIII:C ≥ LLOQ Factane, *n* = 6; Advate, *n* = 18; Kogenate, *n* = 7; Kovaltry, *n* = 4; Afstyla, *n* = 2; Refacto, *n* = 8; Novoeight, *n* = 4; Elocta, *n* = 14
Dosing (IU) per patient	2750 (500–5000)

**Table 2:** Estimates of the population pharmacokinetics parameters.

Parameter	Value (RSE %)	Median of Bootstrap ^†^ (95% CI)
**Fixed Effects**		
Cl (mL·h^−1^)	204 (3.25)	203 (188–218)
βEHL *	−0.394 (10.1)	−0.393 (–0.473–−0.313)
βAge **	−0.214 (21.1)	−0.211 (–0.306–−0.115)
βWeight	0.75 FIX	-
V1 (mL)	2640 (2.04)	2630 (2510–2749)
βWeight ***	0.827 (4.75)	0.833 (0.752–0.917)
Q (mL·h^−1^)	135 (20.5)	136 (54–241)
V2 (mL)	339 (10.7)	327 (221–433)
**Between-Subject Variability**		
ω Cl (%)	34.9 (5.13)	34.6 (30.9–38.6)
ω V1 (%)	23.2 (6.4)	23.0 (18.9–27.2)
ω V2 (%)	46.8 (17.4)	50.0 (19.4–84.4)
**Residual Variability**		
a (constant, IU·mL^−1^)	0.00868 (8.88)	0.00868 (0.00633–0.0112)
b (proportional, %)	10.8 (6.6)	10.6 (7.4–13.8)
**Correlations**		
Corr (V1, Cl)	0.599 (9.75)	0.601 (0.457–0.743)

Abbreviations are as follows: RSE, relative standard errors; CI, confidence interval; Wald test: * *P*-value < 2.2 × 10^−16^; ** *P*-value = 2.4 × 10^−6^; *** *P*-value < 2.2 × 10^−16^. ^†^ from 1000 bootstrap resampling. ${CL}_{i}={CL}_{pop}\times {\left(\frac{{Age}_{i}}{30}\right)}^{-0.214}\times {\left(\frac{{weight}_{i}}{64}\right)}^{0.75}\times e^{-0.394}$; ${V1}_{i}={V1}_{pop}\times {\left(\frac{{weight}_{i}}{64}\right)}^{0.827}$.

## Supplementary Materials

The following are available online at https://www.mdpi.com/1999-4923/12/4/380/s1, Figure S1: Log-Log scale Observed FVIII:C versus individual predictions split by type APTT reagent. Figure S2: Variability comparison of half-lives between final model (EHL on Cl) and final model’ (EHL replaced by Structure covariate on Cl). Table S1: Summary of covariates model building, Table S2: Summary of impact on Between Subject Variability by including ABO, vWF and FFM, Table S3: Characteristics of patients who switched (n = 44) and half-life ratio.
