# Supplementary material for: Real Life Population Pharmacokinetics Modelling of Eight Factors VIII in Patients with Severe Haemophilia A: Is It Always Relevant to Switch to an Extended Half-Life?
Source: Pharmaceutics. 2020 Apr 21;12(4):380. doi: 10.3390/pharmaceutics12040380 (PMC7238177; doi:10.3390/pharmaceutics12040380)
Supplement: Supplementary file 1 [file pharmaceutics-12-00380-s001.docx]

Supplementary Materials: Real Life Population Pharmacokinetics Modelling of Eight Factors VIII in Patients with Severe Haemophilia A: Is it Always Relevant to Switch to an Extended Half-Life?

Quentin Allard, Zoubir Djerada, Claire Pouplard, Yohann Repessé, Dominique Desprez,
Hubert Galinat, Birgit Frotscher, Claire Berger, Annie Harroche, Anne Ryman, Claire Flaujac, Pierre Chamouni, Benoît Guillet, Fabienne Volot, Jean Szymezak, Philippe Nguyen and
Yoann Cazaubon


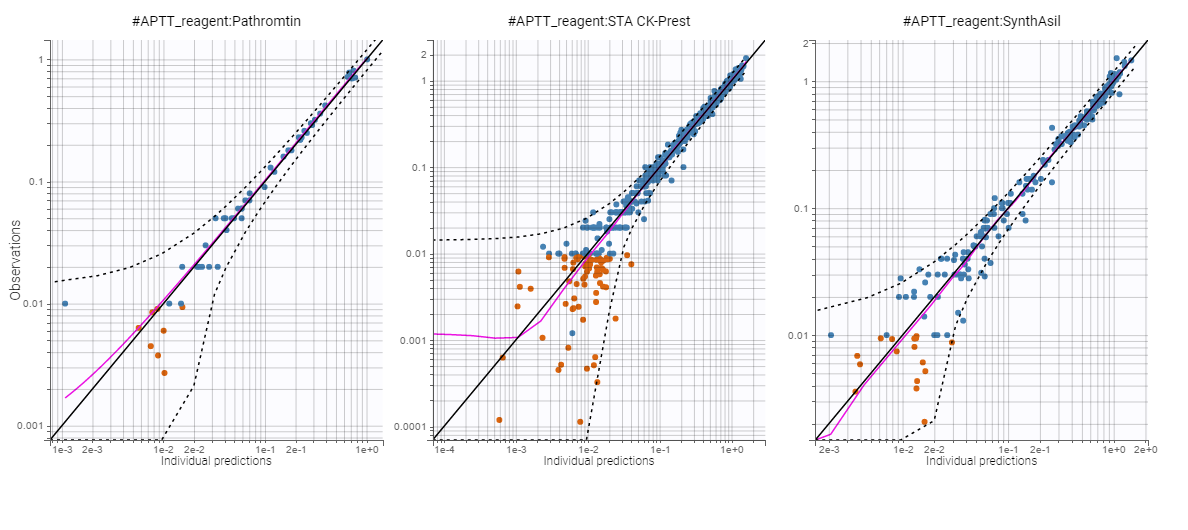


**Figure S1.** Log-Log scale Observed FVIII:C versus individual predictions split by type APTT reagent. The blue dots represent the observed FVIII:C. The orange dots represent the observed FVIII:C BLQ.

**
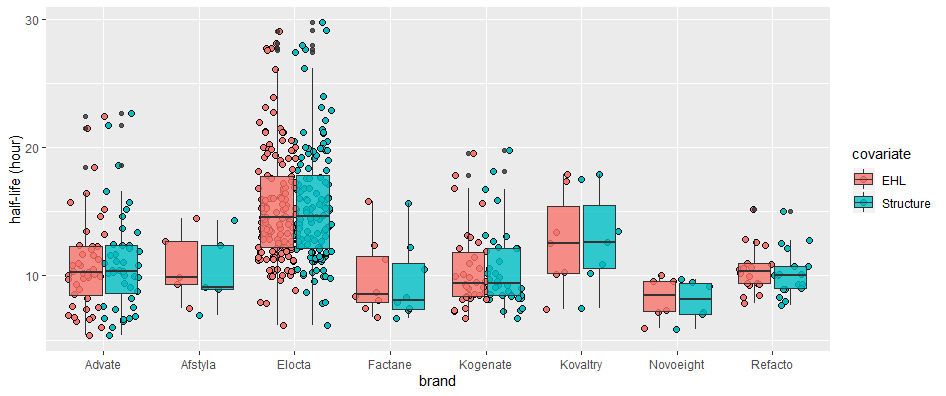
**

**Figure S2.** Variability comparison of half-lives between final model (EHL on Cl) and final model’ (EHL replaced by Structure covariate on Cl).

**Table S1.** Summary of covariates model building.

| **Model** | **Number of fixed effects** | −**2LL** | **BIC** | **ΔBIC** | **RSE of all parameters** |
| --- | --- | --- | --- | --- | --- |
| **1/ Basic model (1 cmt)** | 2 | −1189 | −1156 |  | < 30% |
| **2/ Basic model (2 cmt)** | 4 | −1314 | −1259 |  | < 30% |
| 3/ 2 cmt and no random effect on Q | 4 | −1324 | −1274 | −15 | < 30% |
| 4/ idem 3 and correlation (V1,Cl) | 4 | −1406 | −1351 | −92 | < 30% |
| 5/ idem 4 and age on Cl and weight on V1,Cl | 7 | −1660 | −1588 | −329 | <30% |
| **6/ idem 5 and EHL on Cl** | **8** | −**1765** | −**1687** | −**428** | **< 30%** |
| 7/ idem 5 and Structure | 10 | −1771 | −1683 | −424 | < 30% |
| 8/ idem 5 and Brand | 15 | −1784 | −1673 | −414 | > 50% for 5p |

Abbreviations are as follows: −2LL = −2 x loglikelihood; ΔBIC = BIC (model step) – BIC (basic model, 2cmt); Cmt, Compartment; RSE, Relative standard error; BIC, Bayesian information criterion; EHL, extended half-life; p, parameters.

**Table S2.** Summary of impact on Between Subject Variability by including ABO, vWF and FFM.

|  | **Model** | **BIC** | **ΔBIC** | **ω Cl(%)** | **ω V1(%)** |
| --- | --- | --- | --- | --- | --- |
| ***ABO, n = 207 patients*** | **1/ Basic model (2 cmt) with no random effect on Q and correlation (V1, Cl)** | **−1118** | **-** | **45.4** | **41.1** |
|  | 2/ idem 1 and age, EHL on Cl and weight on V1,Cl | −1339 | −221 | 35.5 | 22.8 |
|  | 3/ idem 2 and ABO group on Cl | −1360 | −242 | 32.4 | 22.3 |
|  | **4/ idem 3 with deletion of age on Cl** | **−1364** | −**246** | **32.5** | **22.9** |
| ***vWF, n = 128 patients*** | **1/ Basic model (2 cmt) with no random effect on Q and correlation (V1, Cl)** | −**703** | **-** | **45.5** | **41.1** |
|  | 2/ idem 1 and age, EHL on Cl and weight on V1,Cl | −895 | −192 | 34.9 | 23.4 |
|  | 3/ idem 2 and vWF group on Cl | −967 | −264 | 29.5 | 23.5 |
|  | **4/ idem 3 with deletion of age on Cl** | −**972** | −**269** | **28.8** | **22.8** |
| ***FFM, n = 197 patients*** | **1/ Basic model (2 cmt) with no random effect on Q and correlation (V1, Cl)** | −**816** | **-** | **46.6** | **46.2** |
|  | 2/ idem 1 and age, EHL on Cl and weight on V1,Cl | −1038 | −222 | 37.2 | 24.8 |
|  | **3/ idem 2 but replacement weight by FFM** | −**1052** | −**236** | **36.7** | **22.7** |

Abbreviations are as follows: ΔBIC = BIC (model step) – BIC (basic model, 2cmt); BIC, Bayesian information criterion; EHL, extended half-life; FFM, Fat Free Mass; vWF, von Willebrand Factor.

**Table S3.** Characteristics of patients who switched (n = 44) and half-life ratio.

| **Patients characteristics** | **Median (min – max)** |
| --- | --- |
| Age (year) | 32.5 (4 - 77) |
| Weight (kg) | 59.5 (16 - 88) |
| Haemophilia status | Only severe, n = 44 |
| Delay between PK switching | 1 week (3 days – 3 months) |
| **brand** | **Ratio (EHL/SHL), median** |
| Advate (n = 16) | 1.58 |
| Factane (n = 1) | 0.98 |
| Kogenate (n = 17) | 1.34 |
| Novoeight (n = 1) | 1.75 |
| Refacto (n = 9) | 1.58 |
